# Genetic sensitivity to emotional cues, racial discrimination and depressive symptoms among African–American adolescent females

**DOI:** 10.3389/fpsyg.2015.00854

**Published:** 2015-06-22

**Authors:** Jessica M. Sales, Jennifer L. Brown, Andrea L. Swartzendruber, Erica L. Smearman, Gene H. Brody, Ralph DiClemente

**Affiliations:** ^1^Department of Behavioral Sciences and Health Education, Rollins School of Public Health, Emory UniversityAtlanta, GA, USA; ^2^Department of Psychological Sciences, Texas Tech UniversityLubbock, TX, USA

**Keywords:** racial discrimination, genetic sensitivity, depressive symptoms, adolescents, *5-HTTLPR*

## Abstract

Psychosocial stress, including stress resulting from racial discrimination (RD), has been associated with elevated depressive symptoms. However, individuals vary in their reactivity to stress, with some variability resulting from genetic differences. Specifically, genetic variation within the linked promoter region of the serotonin transporter gene (*5-HTTLPR)* is related to heightened reactivity to emotional environmental cues. Likewise, variations within this region may interact with stressful life events (e.g., discrimination) to influence depressive symptoms, but this has not been empirically examined in prior studies. The objective of this study was to examine whether variation in the *5-HTTLPR* gene interacts with RD to predict depressive symptoms among a sample of African–American adolescent females. Participants were 304 African–American adolescent females enrolled in a sexually transmitted disease prevention trial. Participants completed a baseline survey assessing psychosocial factors including RD (low vs. high) and depressive symptomatology (low vs. high) and provided a saliva sample for genotyping the risk polymorphism *5-HTTLPR* (*s* allele present vs. not present). In a logistic regression model adjusting for psychosocial correlates of depressive symptoms, an interaction between RD and *5-HTTLPR* group was significantly associated with depressive symptomatology (AOR = 3.79, 95% CI: 1.20–11.98, *p* = 0.02). Follow-up tests found that high RD was significantly associated with greater odds of high depressive symptoms only for participants with the *s* allele. RD and *5-HTTLPR* status interact to differentially impact depressive symptoms among African–American adolescent females. Efforts to decrease depression among minority youth should include interventions which address RD and strengthen factors (e.g., coping, emotion regulation, building support systems) which protect youth from the psychological costs of discrimination.

## Introduction

Across a wide range of health indicators, including mental health outcomes, dramatic racial and socioeconomic health disparities exist for African–American adolescents in the United States (Williams et al., 2010). A variety of factors have been examined to elucidate what drives these persistent disparities. A burgeoning body of literature indicates that racial discrimination (RD) may be a key factor associated with increased risk for many negative health outcomes, including both poorer physical (e.g., coronary artery calcification, sexually transmitted infections, and low birth-weight infants) and mental health outcomes (e.g., increased rates of internalizing and externalizing conditions; Lewis et al., 2006; Brondolo et al., 2008; Dominquez et al., 2008; Pascoe and Smart Richman, 2009; Williams and Mohammed, 2009; Rosenthal et al., 2014). Young African–American women in particular also experience high rates of depressive symptoms relative to their non-minority peers (Khan et al., 2009). For example, 19.3% of African–American adolescent women in the National Longitudinal Study of Adolescent Health endorsed recent and chronic depressive symptoms relative to 13.0% of White adolescent women (Khan et al., 2009). However, not everyone experiencing elevated stressors, including RD stress, evidences negative mental health outcomes. Thus, the primary purpose of this study was to examine the extent to which genetic variation moderates the association between RD and high levels of depressive symptomatology among a sample of adolescent African–American women. In other words, are some adolescent African–American women more affected than others by RD thereby resulting in higher levels of depressive symptoms?

Racial discrimination is defined as dominant group members’ actions that have a differential and negative effect on subordinate racial/ethnic groups (Williams et al., 2003). Previous research has indicated that 91% of pre-adolescent African–Americans reported one or more experiences of race-related discrimination in their lifetime (Gibbons et al., 2004). Similarly, another study found that 77% of African–American youth reported experiencing one or more discriminatory events in the prior 3 months (Prelow et al., 2004). Specific to African–American female adolescents, Guthrie et al. (2002) found that 52% reported at least one exposure to RD in the past year (Guthrie et al., 2002). The Integrative Model for the Study of Developmental Competencies in Minority Children (Integrative Model) by Garcia Coll et al. (1996) proposes that individuals in American society are stratified based upon social position factors (e.g., race, social class, and gender), and social positions are influenced by RD. Because RD is embedded within American society it is a normative and chronic exposure for African–American children and adolescents (Garcia Coll et al., 1996). Thus, RD is a pervasive challenge in the lives of adolescent African–American females, and has been posited by Thoits (1991) and more recently, by Jones (2000) to be a stressor that, if internalized (i.e., internalized racism), threatens the central parts of an individual’s identity, thereby adversely affecting one’s mental health.

A growing body of research suggests that RD is especially harmful to the mental health of African–American youth. Several cross-sectional studies have demonstrated that experiences with RD are associated with lower self-esteem, increased anger, and increased anxiety and depressive symptoms among African–American adolescents (Prelow et al., 2004; Seaton et al., 2008; Gaylord-Harden and Cunningham, 2009). Many of these associations have been found in longitudinal studies as well. Specifically, racial discriminatory experiences are related to decreased self-esteem and increased conduct problems and depressive symptoms among African–American youth (Brody et al., 2006, 2011; Greene et al., 2006; Gibbons et al., 2007; Neblett et al., 2008; Estrada-Marinez et al., 2012).

Depression is one of the most common psychiatric issues affecting adolescents. At any given time, ∼15% of children and adolescents exhibit some symptoms of depression, while 5% of 9- to 17-year-olds meet the criteria for a major depressive disorder (Birmaher et al., 1996; Shaffer et al., 1996). Specific to adolescents, the incidence of depressive disorders markedly increases after puberty. Moreover, by 14 years of age, depressive disorders are more than twice as common in females as in males (Angoid et al., 1999). Female adolescents, a group disproportionately affected by depression, experience heightened interpersonal, relationship stress relative to their male peers (Rudolph, 2002; Hampel and Petermann, 2006; Teva et al., 2010). Added to this, African–American adolescents encounter greater chronic, contextual or environmental stressors relative to their non-minority peers (Copeland-Linder et al., 2011) such as RD (Clark et al., 1999; Sellers et al., 2006; Gaylord-Harden and Cunningham, 2009), among others. Thus for African–American young women in particular, elevated exposure to chronic psychosocial and environmental stressors such as interpersonal stress and RD have been posited as core constructs that may underlie increased vulnerability to depressive disorders and heightened depressive symptoms (Estrada-Marinez et al., 2012). Despite this well-documented association between RD and increased depressive symptoms, surprisingly few studies have examined factors which moderate the association between RD and depressive symptomotology among African–American adolescent females (Seaton et al., 2014), and few, to our knowledge, have taken into account other common sources of stress also associated with depressive symptoms among young women (e.g., interpersonal stress and abuse history; Estrada-Marinez et al., 2012).

Neuroscientists have noted that some individuals are particularly reactive to emotional environmental stimuli, such as exposure to racial discriminatory events. Functional magnetic resonance imaging (fMRI) studies suggest the amygdala, a region of the brain critical for emotional processing and especially important for detection and processing of anxiety and fear-related information, is affected by genetic variability in the promoter of the *5-HTT* gene (*5-HTTLPR;* Nordquist and Oreland, 2010). The *5-HTT* gene is a key regulator of serotonergic neurotransmission, localized to 17p13 and consisting of 14 exons and a single promoter. The common polymorphism in the promoter region results in two variants, a short and a long allele, with the short allele resulting in lower serotonin transporter availability. Although there has been continued debate about the extent to which *5-HTTLPR* moderates the association between stress and depression (see Risch et al., 2009 and Karg et al., 2011 for recent meta-analyses on the topic), relative to those without a short allele, individuals with at least one copy of the short (*s*) allele of the *5-HTTLPR* who have also experienced stressful life events have been suggested to have higher rates of depressive disorders or depressive symptoms.

Aside from this conceptualization of the role of *5-HTTLPR* in emotion-related processing, other studies have found that individuals with at least one copy of the *s* allele of the *5-HTTLPR* have increased amygdala activation to fearful stimuli in facial expression recognition tasks and enhanced amygdala reactivity to punishment cues in the environment (Hariri et al., 2002, 2003; Battaglia et al., 2005; Hariri et al., 2006). Further, carriers of at least one copy of the *s* allele also display hyperactive amygdala response to non-emotional and neutral cues (Heinz et al., 2007; Munafo et al., 2008), direct preferential attention toward threat-related stimuli, and also have difficulty disengaging from such stimuli (Osinsky et al., 2008; Beevers et al., 2009).

Taken together, neuroscience research suggests that carrying the *s* allele may prompt enhanced emotional arousal to threatening and stressful environmental events, resulting in higher levels of depressive symptoms among those with high levels of stress exposure, particularly chronic environmental stressors (e.g., RD). In other words, these findings suggest that African–American adolescent females who experience high levels of RD AND who are also predisposed toward heightened emotional reactivity to environmental events by having at least one copy of the *s* allele of the *5-HTTLPR* should evidence greater levels of depressive symptoms than those without the genetic sensitivity or with lower levels of exposure to RD.

The purpose of the present study was to examine whether variation in the *5-HTTLPR* gene interacts with level of RD to predict depressive symptoms among a sample of adolescent African–American females, while accounting for relevant factors such as coping and social support and stressors (abuse history and interpersonal stress). We hypothesized a gene by environment (G × E) interaction effect in which the association between high levels of RD and high levels of depressive symptoms would be more evident among those individuals carrying at least one copy of the *s* allele of the *5-HTTLPR* polymorphism.

## Materials and Methods

### Description of Parent Study Recruitment

This study is a secondary analysis of data collected as part of a randomized STD/HIV prevention trial specially designed and tailored for adolescent African–American female youth (HORIZONS; DiClemente et al., 2014). From July 2005 to June 2007 African–American adolescent females were recruited from three reproductive health clinics (an adolescent reproductive health clinic in a public hospital, a community located reproductive health clinic, and a county health department STD clinic in Atlanta, GA) to participate in an STD/HIV prevention trial. The adolescent clinic serves ∼2000 clients annually, the majority of clients attending the clinic are ages 16–17, over 90% of clinic attendees are sexually active and the vast majority (over 85%) is African-American. The community-located clinic serves ∼3,300 clients per year; around 80% of female clients are 14-29 years, and ∼50% are African–American. The county health department sexually transmitted disease (STD) clinic serves predominately African–American clients (over 80%), with ∼5,000 adolescent visits to their STD/HIV/AIDS Program annually. The median age of the teens attending the clinic for STD/HIV-related care was about 16 years, and females outnumber males by about two to one.

The purpose of the trial was to assess whether a supplemental treatment delivered after intervention workshop participation (via phone calls) enhanced maintenance of a modified efficacious STD/HIV behavioral intervention (HORIZONS; DiClemente et al., 2014). A young African–American woman recruiter approached adolescents in the clinic waiting area, described the study, solicited participation, and assessed eligibility. Eligibility criteria included self-identifying as African–American, being 14–20 years of age, and reporting at least one instance in the past 6 months of vaginal intercourse without a condom. Young women were excluded from the study if they were married, pregnant, or attempting to become pregnant. Those meeting inclusion criteria and interested in participating returned to the clinic to complete informed consent procedures, baseline assessments, and randomization to trial conditions. Written informed consent was obtained from all young women. Parental consent was waived for those younger than 18 due to the confidential nature of clinic services. Of the eligible individuals, 94% (*N* = 701) enrolled in the study, completed baseline assessments and were randomized to study conditions. Participants were compensated $75 for the baseline visit. The Emory University Institutional Review Board approved all study protocols.

### Procedures and Measures Relevant for the Current Study

As part of the parent study’s procedures, participants completed an audio computer assisted self-interviews (ACASI) at baseline, prior to randomization and intervention participation. The baseline ACASI data allowed for assessment of all variables included in this study such as sociodemographics, coping, social support, abuse history, interpersonal stress, RD, and depressive symptoms. In addition to the baseline ACASI survey completed as part of the parent study, this analysis reports on data from the 304 participants who also consented and provided a saliva sample for DNA analysis^1^.

#### Primary Outcome Variable

##### Depressive symptoms

Depressive symptoms were assessed with a very brief, eight-item version of the Center for Epidemiological Studies-Depression scale (Melchior et al., 1993). The CES-D assesses the presence of depressive symptoms in the past 7 days and has been shown to be a valid measure of depressive symptoms in African–Americans (Radloff, 1991). A total score was first calculated with higher scores indicative of higher depressive symptom levels; the range of possible scores is 8–32. Scores above 15 suggest clinically elevated depressive symptom levels (coded as 1) relative to those with scores below 15 (coded as 0). Cronbach’s α, a measure of the scale’s internal consistency, was 0.91.

#### Primary Predicator Variable

##### Racial discrimination

A 13-item revised version of the Schedule for Racist Events scale (SRE; Landrine and Klonoff, 1996) was used to measure RD. This revised version has been extensively used by other researchers among adolescent and young adult samples (Simons et al., 2003, 2006; Gibbons et al., 2004; Brody et al., 2006). The revised SRE assessed the frequency during the past year, ranging from 1 (never) to 4 (several times), with which the participant experienced specific discriminatory behaviors such as racially based slurs or insults, disrespectful treatment from community members, physical threats, and false accusations from law officials or business employees. The mean score (mean = 20.36, SD = 6.93; median = 20; possible range = 13–52; observed range = 13–45) was used to split the sample into those reporting higher than the average levels of discrimination experiences (scores greater than 20 = 1 “high discrimination”) and those reporting average or lower discrimination experiences (scores 20 or less = 0 “low discrimination”). Cronbach’s α was 0.90.

#### Control Variables

##### Sociodemographic measures

Age was assessed by asking, “How old are you (in years)?” Also, clinic location was included as a control variable as participants were recruited from three downtown Atlanta reproductive health/STD clinics; each serving slightly different populations in regards to SES and education.

#### Psychosocial Correlates

##### Interpersonal stress

A 13-item modified version of the African–American Women’s Stress Scale (Watts-Jones, 1990) was used to measure interpersonal or family stressors. Questions assess the amount of stress an individual feels in various interpersonal relationships or contexts (e.g., relationships with family, partner not being faithful, and isolation from family). Cronbach’s α for the scale was 0.87.

##### History of abuse

Abuse was conceptualized as an index comprising four forms of abuse; emotional, physical, forced vaginal sex or forced anal sex. Abuse history was assessed by asking four questions, “Have you ever been emotionally abused (threatened or called names),” Have you ever been physically abused (hit, kicked, slapped, punched)?”, “Has anyone ever forced you to have vaginal sex when you didn’t want to?”, and “Has anyone ever forced you to have anal sex when you didn’t want to?”. Response choices were yes (1) and no (0). Consistent with the definition used in national surveillance studies (Leeb et al., 2008), a dichotomous composite variable was created in which participants who indicated yes on any of the four items were determined to have a history of abuse, and those who answered no on all items were determined to have no history of abuse.

##### Coping

A 14-item modified version of the COPE scale was used to assess reliance on avoidance-based coping (Carver et al., 1989). Examples of coping behaviors queried were, “I act as though it hasn’t even happened,” or “I admit to myself that I can’t deal with it, and quit trying.” Higher scores indicate more reliance on avoidance-based coping. Cronbach’s α was 0.78.

##### Social support

Social support was assessed with a 12-item scale (Zimet et al., 1988). Responses were coded so that higher scores reflected higher levels of perceived social support by the adolescent. An example item is, “I get the emotional help and support I need from my family.” Cronbach’s α was 0.90.

#### Genotyping

DNA was obtained using Oragene^TM^ DNA kits (Genetek; Calgary, AB, Canada). Participants rinsed their mouths with tap water, and then deposited 4 ml of saliva in the Oragene sample vial. The vial was sealed, inverted, and shipped via courier to a central laboratory in Iowa City, where samples were prepared according to the manufacturer’s specifications. Genotype at *5-HTTLPR* was determined for each sample as previously described (Bradley et al., 2005). Of the sample, 9.2% were homozygous for the short allele (*ss)*, 34.2% were heterozygous (*sl*), and 56.6% were homozygous for the long allele (*ll*). Consistent with prior research (Hariri et al., 2005), genotyping results were used to form two groups of participants: those homozygous for the long allele (*n* = 172) and those with either 1 or 2 copies of the short allele (*n* = 132). Among the 332 participants who provided a saliva sample, 5.12% (*n* = 17) had a “very long” variant of *5-HTTLPR*. Because the activity of this variant on the hypothesized associations has not been well characterized, these youths were excluded from the data analyses.

#### Data Analysis Plan

All analyses were limited to the 304 main trial participants who, in addition to the baseline assessment, consented and provided a valid saliva sample for DNA analysis. Descriptive statistics summarized study variables. In addition, bivariate analyses examined associations between control variables, *5-HTTLPR* group (i.e., *s* allele group vs. *ll* allele group), psychosocial factors associated with managing stressful experiences (i.e., social support and coping), RD group (i.e., low vs. high) and depressive symptoms (i.e., low vs. high). Associations were assessed using Pearson’s correlations and Chi-square analyses. Variables significant at the *p* ≤ 0.10 in bivariate analyses were entered into a multivariable hierarchical logistic regression predicting high depressive symptoms (Hosmer and Lemeshow, 2000), controlling for age and clinic. In the first step, psychosocial correlates were entered into the model. In the second step, RD and *5-HTTLPR* were entered into the model. In the final step, to explore whether the association between discrimination and depressive symptoms differed as a function of *5-HTTLPR* group, an interaction between discrimination group and *5-HTTLPR* group was entered in at this step of the regression model.

## Results

### Sample Description

Descriptive statistics for all measures are presented in **Table 1**. The majority was still in high-school or had only completed some high-school at enrollment (53.9%). Many reported living with their mother only (42.9%), and approximately a quarter had a job for which they were paid. Many of the participants were recruited from a county health department STD clinic (*n* = 154), others were recruited from a reproductive health clinic (*n* = 119), and the remaining participants were recruited from an adolescent reproductive health clinic in a public hospital (*n* = 31). Of the 304 participants in this study 82.2% (*n* = 250) endorsed experiencing a least one of the 13 forms of RD on in the past year.

**Table 1:** Descriptive statistics of the study sample on study variables (*N* = 304).

	Mean	SD
**Sociodemographic**		
Age	18.09	1.40
**Possible psychosocial control variables**		
Interpersonal stress	28.41	13.51
Coping	18.94	4.64
Social support	35.99	5.80
Abuse history (frequency/%)	197	64.8

**Primary predictor variable**	**Frequency**	**%**

High racial discrimination^a^	127	41.8
**Outcome**		
High depressive symptoms^a^	106	34.9

*^a^Rather than means and SD, frequency and percent is displayed*.

### Bivariate Associations Among Study Variables

Pearson correlations or Chi-square tests were conducted among potential control variables, the primary predictor variable (RD), 5-HTTLPR status and depressive symptoms. Only significant (*p* ≤ 0.05), or marginally significant (*p* ≤ 0.10), associations are described. Specific to the control variables, participant age was positively correlated with RD (*r* = 0.15, *p* = 0.01). Also, participants recruited from the health department STD clinic were more likely to report high depressive symptoms (40.9%) than those recruited from the adolescent clinic (32.3%), and the reproductive health clinic (27.7%); χ^2^ = 5.24, *p* = 0.07. Among the psychosocial factors, interpersonal stress (*r* = 0.40, *p* < 0.001), history of abuse (*r* = 0.32, *p* < 0.001), and avoidance-based coping (*r* = 0.27, *p* < 0.001) were each positively correlated with higher depressive symptoms. Participants with high RD were more likely (45.7%) to report high depressive symptoms compared to the those with low discrimination (29.4%); χ^2^ = 19.63, *p* < 0.001. However, participants *5-HTTLPR* status was not significantly related to depressive symptoms (*r* = 0.03, *p* = 0.67), nor were participants with an *s* allele more likely to report higher RD (43.2%) than those in the *ll* allele group (40.7%); χ^2^ = 0.19, *p* = 0.66.

### Multivariable Hierarchical Logistic Regression Predicting Level of Depression Symptoms

Overall, we found that the three step model including the interaction term was significant (see **Table 2**). The interaction between RD and 5*-HTTLPR* group was significantly associated with the probability of being in the high depressive symptom group above and beyond the psychosocial factors. In order to interpret the interaction effect, separate multivariable logistic regression models for level of depressive symptoms were conducted for those possessing one or two copies of the *s* allele and those with the *ll* allele (see **Table 3**). For both those with the *s* and the *ll* genotypes, having higher interpersonal stress and a history or abuse were associated with higher odds for elevated depressive symptoms. However, RD was associated with higher odds of elevated depressive symptoms only among those with the *s* allele, but engagement in avoidance-based coping was associated with higher odds for elevated depressive symptoms among the *ll*-genotype.

**Table 2:** Multivariable hierarchical logistic regression predicting level of depressive symptoms.

				95% CI	
Predictors	β	SE	Odds ratio	Lower	Upper	p
**Step 1**
**Psychosocial correlates**
Interpersonal stress	0.06	0.01	1.06	1.04	1.08	0.001
Coping	0.11	0.03	1.12	1.05	1.20	0.001
Abuse history	1.15	0.36	3.15	1.56	6.35	0.001
**Step 2**
5-HTTLPR group	-0.72	0.40	0.49	0.22	1.06	0.071
Racial discrimination group	-0.28	0.39	0.76	0.35	1.66	0.486
**Step 3**
Discrimination X5-HTTLPR group	1.33	0.58	3.79	1.20	11.98	0.023
Step 1 Xi^2^	82.15					0.001
Step 2 Xi^2^	1.16					0.561
Step 3 Xi^2^	5.31					0.021
Overall model Xi^2^	88.61					0.001

*Age and clinic were controlled for in regression*.

**Table 3:** Multivariable logistic regressions predicting level of depressive symptoms, separately for each *5-HTTLPR* group.

				95% CI		
Predictors	β	SE	Odds ratio	Lower	Upper	*p*
***5-HTTLPR s* allele group**
**Primary predictor**
Racial discrimination group	0.91	0.45	2.48	1.02	6.05	0.045
**Psychosocial factors**
Interpersonal stress	0.06	0.02	1.06	1.02	1.10	0.002
Coping	0.05	0.05	1.05	0.95	1.16	0.358
Abuse history	1.38	0.55	3.93	1.33	11.64	0.014
Overall χ^2^ =	36.05					0.001
***5-HTTLPR ll* allele group**
**Primary predictor**
Racial discrimination group	-0.20	0.43	0.82	0.35	1.90	0.637
**Psychosocial factors**
Interpersonal stress	0.06	0.02	1.06	1.03	1.09	0.001
Coping	0.16	0.05	1.18	1.08	1.29	0.001
Abuse history	0.96	0.49	2.61	1.01	6.76	0.048
Overall χ^2^ =	55.55					0.001


*Age and clinic were controlled for in regressions*.

**FIGURE 1:**
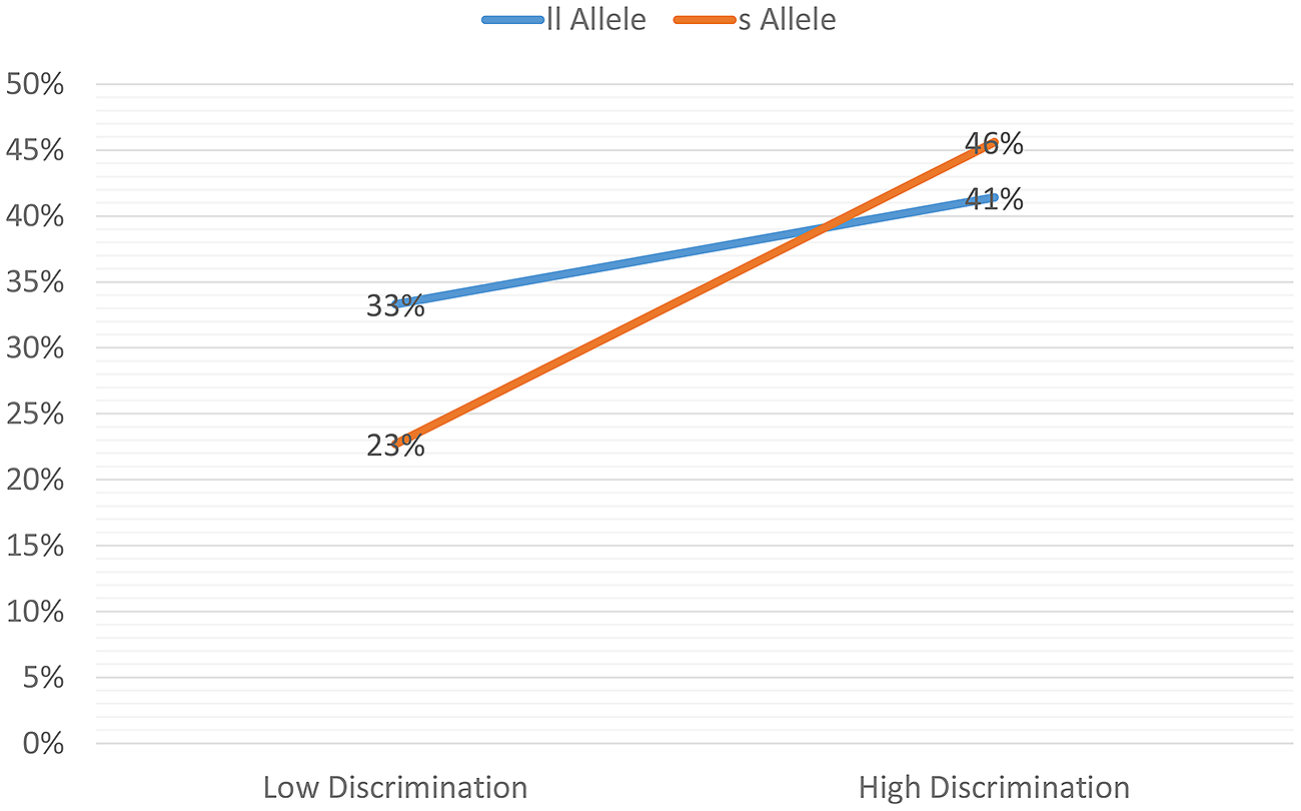
**Percent reporting high depressive symptoms: interaction between discrimination and *5-HTTLPR***.

An additional follow-up test was conducted to further examine the proposed differential susceptibility hypothesis, whereby those with the *s* allele are more sensitive toward and responsive to their environment. A layered Chi-Square test was conducted, with separate Chi-Square tests run by *5-HTTLPR* group (*s* allele group and the *ll* allele group) to determine the association between level of RD (low vs. high) and level of depressive symptoms (low vs. high) for each genetic group (see **Figure 1**). For those youth in the s allele group (*n* = 132), participants with high levels of discrimination experiences were significantly more likely to report high levels of depressive symptoms (45.6%) than those with low levels of discrimination experiences (22.7%); χ^2^ = 7.77, *p* = 0.005. In contrast, among those in the *ll* allele group (*n* = 172), participants with high levels of discrimination experiences were not more likely to report high levels of depressive symptoms (41.4%) than those with low levels of discrimination experiences (33.3%); χ^2^ = 1.17, *p* = 0.28.

## Discussion

Similar to previous reports of RD levels in the U.S., almost all participants in this African–American adolescent female sample reported experiencing RD in the prior year, with ∼41% indicating higher than average experiences of discrimination. This sample also showed high levels of depressive symptoms, with ∼35% reporting levels of depressive symptoms that are potentially clinically significant. In addition to other stressors (interpersonal stress and abuse histories), as expected (Prelow et al., 2004; Seaton et al., 2008; Gaylord-Harden and Cunningham, 2009), RD was significantly associated with depressive symptoms among this all female sample. However, results demonstrated a moderating role of *5-HTTLPR* genotype status in this outcome. Among this sample of young African–American women, high exposure to RD was associated with greater odds of elevated depressive symptoms only for young women with the *s* allele, even when accounting for multiple sources of stress and psychosocial factors, while the link between RD and depressive symptoms was attenuated for those with the *ll* genotype. Among individuals with this genotype, greater reliance on avoidance-based coping behaviors were instead significantly associated with likelihood of elevated depression symptoms.

Adolescence is a period of great developmental change (Crosby et al., 2009) characterized by increasing levels of psychosocial stressors (Compas, 1987). For young women especially, adolescence is a time when many may experience depressive symptoms for the first time (Angoid et al., 1999). African–American adolescents may also encounter additional chronic, contextual or environmental stressors relative to their non-minority peers (Copeland-Linder et al., 2011; Estrada-Marinez et al., 2012). This stressful exposure includes RD, which has been specifically associated with increased risk for elevated depressive symptoms particularly among young women (Clark et al., 1999; Sellers et al., 2006; Gaylord-Harden and Cunningham, 2009). The genetic marker of *5-HHTLPR* as indicated through the neuroscience findings seems to be especially relevant for the detection of fear or threat specific stimuli in the environment (particularly in facial cues). The neuroscience findings that individuals with the *s* allele seem to be hyperaroused and hypersensitive to cues of fear/threat make this marker particularly important in the context of some stressors (like racial discriminatory behaviors such as physical threats, slurs, insults, and it is also relevant for individuals with histories of physical or sexual abuse as well), but it may not be as relevant in other stressful contexts that may not contain the same sort of emotional triggers detected by monitoring others emotional states, such as interpersonal stressors like someone owing you money, or minor daily hassles captured in our interpersonal stress scale. However, this speculation warrants further examination.

In accordance with the differential susceptibility theories (Belsky et al., 2007), we found a link between exposure to high levels of RD and depressive symptoms among youth who carried the one of two copies of the *s* allele of the *5-HTTLPR*. This finding is consistent with neuroimaging studies and prior G × E research involving the *5-HTTLPR* such that female youth who carry the short allele, because of their genetic make-up, may be more reactive to emotional or threating social cues in their environments (e.g., being disrespectfully treated by community members) and are therefore more negatively impacted by experiences of RD than those with the *ll* genotype. The finding that carrying two copies of the long allele may confer protection from depressive symptoms when they experience high levels of discrimination is relevant to research on youth resilience.

The resilience literature has addressed potential reasons why some youth who experience adverse experiences, including exposures to chronic stressors, do not succumb to their negative effects (Luthar, 2006). The present results support recent findings suggesting that genetic status may also contribute to resilience (Rutter and Silberg, 2002; Moffitt et al., 2006; Kim-Cohen and Gold, 2009; Brody et al., 2011). Particularly, the finding that carrying two *l* alleles attenuates the association between discrimination and depressive symptoms suggests a possible emotional self-regulatory mechanism by which genotype contributes to the down regulation of emotions resulting from discriminatory experiences (Simons et al., 2003, 2006; Brody et al., 2011), but this potential mechanistic pathway requires further investigation. Further, the potentially protective effects of the *ll* allele may not be generalizable to all youth, or under all contexts. Similar to Estrada-Marinez et al.’s (2012) findings that not all stressors are equally impactful on externalizing and internalizing outcomes, our results also suggest that young women with the *ll* allele may be resilient in some areas but experience distress in others, such as when they experience certain aspects of interpersonal relationships that may not be perceived as threatening yet cause stress.

Our findings also are in line with Belsky et al. (2007) differential susceptibility hypothesis in which gene-specific variants are speculated to render individuals more susceptible to their surrounding environments, whether those be “good” or “bad” environments. Specifically, we observed that young women who carried the *s* allele, in low discriminatory contexts reported lower levels of depressive symptoms than did *ll* allele carriers (see **Figure 1**), thereby supporting the hypothesis of differential susceptibility. However, we know very little about other positive or protective attributes of these young women’s environments, but this would be an important avenue for further investigation, especially as it may shed light on potential protective factors that could serve as intervention opportunities to decrease adverse mental health outcomes among those adolescent women exposed to high levels of discrimination.

From an intervention perspective, the results suggest that young African–American women seeking sexual health services would benefit from additional resources and skills training to address depressive symptoms and cope with chronic, pervasive stressors including experiences of RD. For example, multiple health-related intervention approaches may benefit from inclusion of content to improve coping and self-management strategies. Specifically, it may be especially beneficial given the high rates of stress (whether from interpersonal relationships or from experiences of discrimination) that health promotion programs for adolescent and young adult African–American females in general include mental-health specific components, such as teaching developmentally appropriate stress-coping skills and cognitive behavioral management skills (Antoni et al., 2001) mindfulness training that would help youth learn relaxation techniques for managing uncontrollable stressors (Bishop, 2002), and strategies for building and accessing social support systems. These components could be integrated into existing STD prevention interventions in the case of this study, or other health promotion programs, to address likely unmet mental health needs of the youth who are experiencing high levels of discrimination or other chronic stressors. Importantly, for some youth (those with the *s* allele) who are particularly reactive to environmental cues of threat, other clinical techniques (e.g., exposure therapy approaches) may be useful to reduce threat sensitivity.

### Limitations

This study is not without limitations. First, the sample consisted of adolescents who were seeking services at sexual health clinics, who met eligibility criteria for the parent study, and who attended the follow-up visit when the genetic sample was collected. Therefore results may not generalize to individuals who do not access similar clinics, who would not meet the eligibility criteria, which included having recent unprotected sex, or who are not likely to return for follow-up. Future research should include a broader sample of youth, as well as include males to extend or replicate these findings. Also, the data employed in this study were only from participants who returned at the 24-month follow-up assessment and provided a saliva sample. It is possible that returning participants may have differed in meaningful ways from those who did not return for follow-up, but we have no way to formally examine this possibility. However, analyses of baseline socioeconomic indicate no significant differences between those who returned for follow-up and those who did not. Additionally, participants who provided DNA samples may have differed from participants who did not provide a specimen. However, we experienced a high rate of participation for the DNA saliva collection (92%), and a comparison of baseline characteristics indicates no observed differences in sociodemographics, psychosocial variables, or behavioral outcomes. Finally, the self-report data are cross-sectional, making it difficult to assess causal relationships, although a strength is that we directly assessed RD in our study.

## Conclusion

Adolescence is a period of life characterized by developmental change and increasing levels of psychosocial stressors (Compas, 1987). For African–Americans, many will encounter additional chronic, contextual or environmental stressors such as exposure to RD relative to their non-minority peers (Copeland-Linder et al., 2011; Estrada-Marinez et al., 2012). However, consistent with neuroimaging studies and prior G × E research involving the *5-HTTLPR*, for some individuals, because of their genetic make-up, they are particularly susceptible to negative psychological consequences resulting from high exposure to RD than others. Given the high rates of depressive symptoms coupled with high number of stressors reported among adolescent African–American (whether from abuse experiences, interpersonal relationships or from experiences of discrimination) it may be advantageous for health promotion programs targeting adolescent and young adult African–American females in general to include mental-health specific components, such as teaching developmentally appropriate stress management and cognitive behavioral skills.

## Conflict of Interest Statement

The authors declare that the research was conducted in the absence of any commercial or financial relationships that could be construed as a potential conflict of interest.

## Footnotes

1 The DNA sample collection was an addition to the main trail’s data collection. Because of this, not every participant was invited to provide a sample if they: (1) had already completed the trial, or (2) did not return for the 24 month follow-up assessment when the sample collection occurred. 363 were asked to provide a sample, and only 31 declined.
